# Issues in the Pharmacokinetics of Trichloroethylene and Its Metabolites

**DOI:** 10.1289/ehp.8691

**Published:** 2006-05-09

**Authors:** Weihsueh A. Chiu, Miles S. Okino, John C. Lipscomb, Marina V. Evans

**Affiliations:** 1 National Center for Environmental Assessment, U.S. Environmental Protection Agency, Washington, DC, USA; 2 National Exposure Research Laboratory, U.S. Environmental Protection Agency, Las Vegas, Nevada, USA; 3 National Center for Environmental Assessment, U.S. Environmental Protection Agency, Cincinnati, Ohio, USA; 4 National Health and Environmental Effects Research Laboratory, U.S. Environmental Protection Agency, Research Triangle Park, North Carolina, USA

**Keywords:** metabolism, pharmacokinetics, physiologically based pharmacokinetic model, risk assessment, trichloroethylene

## Abstract

Much progress has been made in understanding the complex pharmacokinetics of trichloroethylene (TCE). Qualitatively, it is clear that TCE is metabolized to multiple metabolites either locally or into systemic circulation. Many of these metabolites are thought to have toxicologic importance. In addition, efforts to develop physiologically based pharmacokinetic (PBPK) models have led to a better quantitative assessment of the dosimetry of TCE and several of its metabolites. As part of a mini-monograph on key issues in the health risk assessment of TCE, this article is a review of a number of the current scientific issues in TCE pharmacokinetics and recent PBPK modeling efforts with a focus on literature published since 2000. Particular attention is paid to factors affecting PBPK modeling for application to risk assessment. Recent TCE PBPK modeling efforts, coupled with methodologic advances in characterizing uncertainty and variability, suggest that rigorous application of PBPK modeling to TCE risk assessment appears feasible at least for TCE and its major oxidative metabolites trichloroacetic acid and trichloroethanol. However, a number of basic structural hypotheses such as enterohepatic recirculation, plasma binding, and flow- or diffusion-limited treatment of tissue distribution require additional evaluation and analysis. Moreover, there are a number of metabolites of potential toxicologic interest, such as chloral, dichloroacetic acid, and those derived from glutathione conjugation, for which reliable pharmacokinetic data is sparse because of analytical difficulties or low concentrations in systemic circulation. It will be a challenge to develop reliable dosimetry for such cases.

Understanding trichloroethylene (TCE) pharmacokinetics—the dynamic behavior of chemical absorption, distribution, metabolism, and excretion (ADME)—is critical to both the qualitative and quantitative assessments of human health risk from environmental exposures. Qualitatively, pharmacokinetic information can help identify the chemical species that may be causally associated with observed toxic responses. This is particularly important for TCE because many of its toxic effects are thought to be due to metabolites rather than to TCE alone (Caldwell and Keshava 2006). In addition the delineation of inter- and intraspecies pharmacokinetic differences can provide insight into how laboratory animal and epidemiological data may inform overall human health risks and how individual susceptibility may differ. Furthermore, physiologically based pharmacokinetic (PBPK) models can quantify the relationship between external measures of exposure and internal measures of toxicologically relevant dose. Selection of an appropriate dose metric for use in risk assessment depends on both an understanding of the target tissue, active chemical agent, and mode of action (MOA) for a particular toxic effect [see Caldwell and Keshava (2006) and Keshava and Caldwell (2006) for additional MOA discussion] and the reliability of the PBPK models themselves. The state-of-the-science monograph on TCE health risks in *Environmental Health Perspectives* (Scott and Cogliano 2000) included reports on a number of PBPK models for TCE and its metabolites and on applications of these models (Barton and Clewell 2000; Bois 2000a, 2000b; Clewell et al. 2000; Fisher 2000; Rhomberg 2000). The U.S. Environmental Protection Agency (U.S. EPA), in its 2001 draft TCE assessment (U.S. EPA 2001), used TCE PBPK models to address a number of different risk assessment issues, including cross-species pharmacokinetic extrapolation of rodent studies of both cancer and noncancer effects, exposure route extrapolation, and characterization of human pharmacokinetic variability.

In this article we present an updated review of the pharmacokinetics of TCE and its metabolites and the factors affecting PBPK modeling, focusing on information that has emerged since publication of the *Environmental Health Perspectives*’ state-of-the-science monograph in 2000 (Scott and Cogliano 2000). Although some scientific conclusions can be drawn from this updated body of data, speculation as to the effect of these data on the final TCE risk assessment would be premature at this point, given the ongoing National Academy of Sciences consultation discussed in the overview article (Chiu et al. 2006) and the subsequently planned revision of the U.S. EPA TCE risk assessment. Therefore, this mini-monograph is a review of recently published scientific literature in the context of how it informs the key scientific issues believed to be most critical to developing a revised risk assessment. In particular, in the present article we describe the major issues related to the ADME of TCE and its metabolites; discuss PBPK models for TCE and its metabolites, including the four models/parameterizations reviewed or published in 2000 (Bois 2000a, 2000b; Clewell et al. 2000; Fisher 2000) and several recent and ongoing PBPK modeling efforts; and summarize the current challenges to PBPK modeling for application to risk assessment.

## Scientific Uncertainties in the Pharmacokinetics of TCE and Its Metabolites

Lash et al. (2000a) presented a comprehensive review of the ADME of TCE and its metabolites as part of the 2000 state-of-the-science monograph on TCE health risks (Scott and Cogliano 2000), with a summary presented in the U.S. EPA 2001 draft risk assessment (U.S. EPA 2001). Briefly, TCE is rapidly and extensively absorbed via all routes of environmental exposure—ingestion, inhalation, and dermal contact. Once absorbed, TCE is distributed via the circulatory system throughout the body. The majority of TCE taken into the body is metabolized; direct exhalation is the other major route of elimination of the parent compound (Lash et al. 2000a).

**Figure 1:**
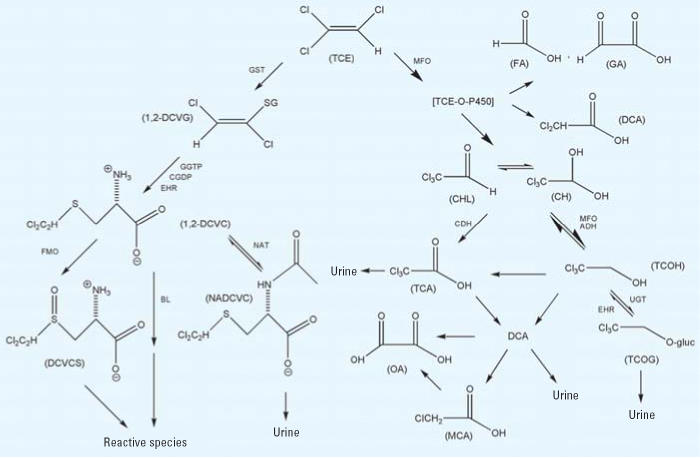
Postulated metabolism scheme for trichloroethylene. Figure adapted from Clewell et al. (2000) and Lash et al. (2000a). For the GSH pathway, metabolism to 1,2-DCVG is shown, but 1,1-DCVG goes through similar steps through 1,1-DCVC to *N*-acetylated and reactive species. Abbreviations: ADH, alcohol dehydrogenase; BL, cysteine conjugate β-lyase; CDH, chloral dehydrogenase (aldehyde oxidase); CGDP, cysteinyl-glycine dipeptidase; CH, chloral hydrate; CHL, chloral; DCA, dichloroacetic acid; DCVC, *S*-dichlorovinyl-l-cysteine; DCVCS, 1,2-DCVC sulfoxide; DCVG, *S*-dichlorovinyl glutathione; EHR, enterohepatic recirculation; FA, formic acid; FMO, flavin-containing monooxygenase; GA, glyoxylic acid; GGTP, γ-glutamyl transpeptidase; GST, glutathione *S*-transferase; MCA, monochloroacetic acid; MFO, mixed-function oxidase (i.e., cytochrome P450); NAT, *N-*acetyltransferase; NADCVC, *N*-acetyl-1,2-DCVC; OA, oxalic acid; TCE-O-P450, oxygenated TCE-cytochrome P450 transition state complex; TCA, trichloroacetic acid; TCOG, trichloroethanol glucuronide; TCOH, trichloroethanol; UGT, uridine diphosphate-glucuronosyltransferase.

A postulated scheme for the pathways of TCE metabolism—adapted from the work of Lash et al. (2000a), Clewell et al. (2000), and recent studies described later in this section—is presented in Figure 1. As shown in the figure, TCE and some of its subsequent metabolites are processed through a number of branching metabolic pathways; therefore, assessment of its pharmacokinetics is complicated. Metabolism of TCE itself occurs through two main irreversible pathways: oxidation via the microsomal mixed-function oxidase (MFO) system [i.e., cytochrome P450s (CYPs)] primarily to chloral/chloral hydrate (CHL/CH; see the discussion below regarding TCE-oxide) and conjugation with glutathione (GSH) by glutathione *S*-transferases (GSTs) to *S*-dichlorovinyl glutathione (DCVG). For TCE oxidation, CYP2E1 is thought to be most important *in vivo.* Subsequent important metabolic branch points include the production of trichloroethanol (TCOH) and trichloroacetic acid (TCA) from CHL/CH; the production of TCOH glucuronide (TCOG) and regeneration of CHL/CH from TCOH, and the *N*-acetylation versus bioactivation of *S*-dichlorovinyl-l-cysteine (DCVC). The major metabolites detected in blood and urine after TCE exposure are TCOH/TCOG and TCA, and some evidence suggests the direct production of TCA from TCOH. Further metabolism of these species, if any, is not well characterized; the downstream metabolites have not been consistently detected *in vivo*. The enzyme families involved in many of these transformations are thought to be known. Little, if any, information is available, however, regarding the specific isoforms involved or the differences in their concentrations or proportions across species, individuals, or organs.

Important issues relating to understanding TCE pharmacokinetics are discussed below. Particularly important for risk assessment is whether sufficient information exists both within and across species to quantify rates of TCE metabolism, flux through different metabolic pathways, and distribution and excretion of the metabolites.

### Enterohepatic recirculation of TCA and TCOH

In the liver, chemicals can be secreted into the bile and transported into the gut, where they are reabsorbed into the portal blood, thereby increasing the effective half-life for systemic clearance. Two of TCE’s oxidative metabolites, TCA and TCOH, have been measured in the bile of rats (Stenner et al. 1997). Bile-cannulated rats showed different blood concentration profiles, providing *in vivo* evidence for such recirculation. A PBPK model based on this work (Stenner et al. 1998) included enterohepatic recirculation (EHR) and showed a reasonable match to rat concentration profiles after oral doses of TCE (in 2% Tween 80) and intravenous doses of TCA and TCOH. Difficulties exist in extrapolating the rat data to other species because biliary excretion does not scale uniformly, as shown in studies with therapeutics (Mahmood and Sahajwalla 2002).

The significance of recirculation on important dose metrics is uncertain because existing PBPK models have generally shown reasonable fits to blood and urine data without recirculation. For example, even though Clewell et al. (2000) implemented recirculation structurally, reabsorption in the gut was set to zero for comparison with most data. Bois (2000a) noted, however, that urinary excretion data for TCOG in mice was not well fit by the Fisher model, which did not include recirculation. Overall, evaluation of the model fit and sensitivity of TCA- and TCOH-related dose metrics with and without EHR has not been reported.

### Diffusion-limited tissue distribution in fat and liver

Most of the PBPK models for TCE assume flow-limited distribution of chemicals to the organ compartments, a representation that assumes compartments are well mixed and that the chemical concentration in the blood leaving the tissue has reached equilibrium with the concentration in the tissue. However, the fat and liver are known to be heterogeneous tissues (e.g., Andersen et al. 1997) and important to the distribution and metabolism of volatile organics, respectively.

Bois (2000a) reported that the measured adiposity of the individual subjects from Fisher et al. (1998) did not correlate well with the posterior estimates for the model parameter for percentage body weight as fat. Bois suggested one possible explanation in that the pharmacokinetic compartment for fat may not be well estimated by external adiposity measurements, but model error cannot be excluded. Albanese et al. (2002) suggested that a compartmental model for fat does not adequately capture the concentration profile of TCE in adipose tissue. Consequently, they developed an axial dispersion model designed to account for physiologic heterogeneities. These authors compared the perfusion-limited, diffusion-limited, and axial dispersion models and concluded that the axial dispersion model is best able to capture the physiologic heterogeneities of adipose tissue and their expected effects on TCE adipose concentrations.

Keys et al. (2003) recently developed a PBPK model for TCE parent kinetics in rats and mice that used two-compartment descriptions of the fat and liver to better fit parent compound time-courses in those tissues. For the fat, fat blood and fat tissue were both explicitly modeled, with transport between them changed from flow limited to diffusion limited. The liver was divided into a “shallow” compartment (assumed to be the site of metabolism) and a “deep” compartment, with transport between them via diffusion. Although the deep compartment was proposed to represent the lipid portion of the liver, the authors noted that the physiologic basis for a deep liver compartment was not understood. Keys et al. (2003) concluded that TCE parent concentrations are better simulated by this more complex model and that although other dose metrics were not evaluated, metabolite concentrations would not be expected to be significantly changed. Lipscomb et al. (1998, 2003a) used a flow-limited PBPK model to simulate the variability in hepatic CYP2E1 content that was measured *in vitro* (Lipscomb et al. 1997, 2003b; Snawder and Lipscomb 2000). The flow-limited model indicated that the flux of TCE oxidation was not sensitive to enzyme content but was instead limited by hepatic blood flow. However, the relative contributions of transport and metabolism in the liver may change with a diffusion-limited description. The importance of more complex descriptions of both liver and fat needs to be determined because the liver is considered to be a target organ and the fat can store TCE.

### Plasma binding of TCA and dichloroacetic acid

The binding of chemicals to proteins in plasma affects their availability to other tissues and their effective half-life in the body. The TCE metabolites TCA and dichloroacetic acid (DCA) bind to plasma proteins. Lumpkin et al. (2003), Schultz et al. (1999), Templin et al. (1995), and Yu et al. (2000) all measured TCA binding in various species and at various concentration ranges. Of these, Templin et al. (1995) and Lumpkin et al. (2003) measured levels in humans, mice, and rats. Lumpkin et al. (2003) studied the widest concentration range, spanning reported TCA plasma concentrations from experimental studies. However, these data are not entirely consistent among researchers; 2- to 3-fold differences are noted in some cases, although some differences existed in the rodent strains and experimental protocols used.

Schultz et al. (1999) also measured DCA binding in rats at a single concentration of about 100 μM and found a binding fraction of < 10%. However, these data are not greatly informative for TCE exposure in which DCA levels are significantly lower, and limitation to a single concentration precludes fitting to standard binding equations from which the binding at low concentrations could be extrapolated. Because of the observed species differences in TCA binding, direct extrapolation of the DCA rat binding data to other species may not be accurate.

Stenner et al. (1998) and Clewell et al. (2004) have incorporated plasma binding of TCA in PBPK models of TCE. The authors assume that the tissue-bound/free ratio is in equilibrium with blood, but only the free fraction is available for exchange with tissues. The binding equilibrium assumption requires that the time scales of binding are fast relative to the other ADME processes but slower than the tissue perfusion time scale. However, existing studies have not reported the time scale of DCA or TCA binding kinetics. Evaluation of the impact of the binding uncertainties associated with the kinetics and differing experimental observations on the PBPK model dose metrics has not been reported.

### DCA formation, pharmacokinetics, and the role of trichloroethylene oxide (epoxide)

Recent data suggest that DCA is one of the TCE metabolites involved in rodent liver tumor induction [Bull 2000; see also discussion in Caldwell and Keshava (2006)]. As noted by Lash et al. (2000a), although DCA has been reported *in vivo* after TCE exposure in both mice and humans, considerable uncertainty remains in the levels actually produced because of known analytical limitations in the available DCA measurements. In addition the multiple hypotheses regarding how DCA may be formed and the self-inhibition of its metabolism complicate interpretation of these data.

Detection of DCA production *in vivo* after TCE administration has been complicated by reported problems with analytical methodologies that have led to artifactual formation of DCA *ex vivo* when samples contain significant amounts of TCA (Ketcha et al. 1996). After the discovery of these analytical issues, Merdink et al. (1998) reevaluated the formation of DCA from TCE, TCOH, and TCA in mice, with particular focus on the hypothesis that DCA is formed from dechlorination of TCA. They were unable to detect blood DCA in naive mice after administration of TCE, TCOH, or TCA. Several other *in vivo* studies continued to report circulating DCA in mice after TCE exposure (Abbas and Fisher 1997; Greenberg et al. 1999). Fisher et al. (1998) reported the results of a controlled human exposure study in which DCA was detected in some but not all human blood samples. For all these studies, the extent to which analytical artifacts of DCA remain is unclear, so these data may be useful only for upper bounds. However, even low DCA levels may have toxicologic significance.

Lash et al. (2000a) discussed two potential sources of DCA formation, from TCOH and from dechlorination of TCA. [DCA does not appear to be formed by gut microflora (Moghaddam et al. 1996, 1997).] Merdink et al. (2000) investigated dechlorination of TCA and reported trapping a DCA radical with the spin-trapping agent phenyl-*tert*-butyl nitroxide, identified by gas chromatography/mass spectroscopy, in both a chemical Fenton system and rodent microsomal incubations with TCA as substrate. On the other hand, the work reviewed by Guengerich (2004) has suggested that a source of DCA may be through a TCE oxide (epoxide) intermediary. Although oxidation of TCE by CYPs results predominantly in CHL (in equilibrium with CH) (Lash et al. 2000a), previous work of Miller and Guengerich (1983) had reported evidence of formation of the epoxide as an independent oxidative pathway (i.e., not leading to formation of CHL). In addition Cai and Guengerich (1999) reported that a significant amount (about 35%) of DCA is formed from aqueous decomposition of TCE oxide via hydrolysis in an almost pH-independent manner. Because this reaction forming DCA from TCE oxide is a chemical process rather than a process mediated by enzymes, and because evidence suggests that some epoxide was formed from TCE oxidation, Guengerich (2004) noted that DCA would be an expected product of TCE oxidation.

Single doses of DCA are rapidly metabolized by GST-ζ, but self-inhibition of this metabolic pathway has been observed over repeated exposures and has been quantified in rodents (Barton et al. 1999; Gonzalez-Leon et al. 1997, 1999; Schultz et al. 2002) and in humans (Curry et al. 1991). Keys et al. (2004) developed a PBPK model for DCA and its self-inhibition of metabolism in rats and mice. They reported that assuming a second GST-ζ–independent clearance pathway substantially improved the fit to DCA time courses, with the relative flux through this pathway increasing with DCA dose because of self-inhibition of GST-ζ; however, there appears to be no other evidence for such a pathway. The incorporation of DCA models that include representations of the metabolism and formation pathways into TCE models will allow for evaluation of DCA-related dose metrics after TCE exposure, but a human PBPK model for DCA has not yet been developed.

### Pathways of glutathione conjugation and subsequent metabolism

As discussed by Caldwell and Keshava (2006), some GSH metabolites of TCE are specific and potent renal toxicants *in vitro* and *in vivo*, with effects depending on both exposure concentration and duration. However, Lash et al. (2000b) noted that the processing of GSH conjugates is complex and poorly understood relative to the processing of oxidative metabolites, with a number of different metabolites both locally produced in the kidney and transported to the kidney from the liver. In particular, quantitative uncertainties exist in the production of GSH conjugates from TCE, their interorgan transport, and their subsequent processing through bioactivation and detoxification.

The first stable product of the conjugation of TCE is DCVG, which is subsequently processed to DCVC. Metabolic rate constants have been measured *in vitro* for the conjugation of TCE with GSH (Lash et al. 1999a), but data on the specific GST form/subunit responsible are limited (see below), and no reliable protein recovery data exist to serve as the basis for an *in vitro* to *in vivo* extrapolation of metabolic rate constants for GSH conjugation. Without such data, extrapolation of these metabolic rate constants for application in PBPK modeling–based approaches is highly uncertain. Interestingly, however, the formation rate of DCVG measured in isolated hepatocytes was similar in order of magnitude to that measured for oxidative metabolites. Specifically, Lipscomb et al. (1998) reported the *V*_max_ for oxidation to range from 6 to 41 (mean 16) nmol/hr/million cells and *K_m_* values of 81–510 (mean, 266) ppm in headspace (*n* = 6). For the GSH pathway, using similar experimental procedures, Lash et al. (1999a) measured rates of DCVG production at concentrations from 25 to 10,000 ppm in headspace. Although rate constants were not reported, they show maximal rates averaging around 10 nmol/hr/million cells at concentrations around and above the oxidation *K_m_* (250–7,000 ppm in headspace) (*n* = 3) and an average rate of around 6 nmol/hr/million cells (i.e., around half the maximal rate) at 50 ppm (*n* = 3).

In addition a number of *in vivo* studies provide evidence for the GSH pathway being active in humans. Bernauer et al. (1996) and Birner et al. (1993) reported measuring the urinary metabolites of DCVC such as *N*-acetyl DCVC in humans, which provided an indicator of GSH conjugation, at least through the *N*-acetyltransferase (NAT) detoxification pathway. Further evidence was found by Lash et al. (1999b), who detected DCVG in the blood of human volunteers exposed to TCE. However, the subsequent conjugation product DCVC was not detected in blood, and the corresponding mercapturates were detected only sporadically in urine. Bloemen et al. (2001) measured GSH pathway metabolites in the urine of human volunteers and occupationally exposed workers. Although Bloemen et al. (2001) reported that levels were below detection limits in all cases, their results appear to be consistent with those of Bernauer et al. (1996). In particular, based on their detection limits, Bloemen et al. (2001) place an upper bound of 0.05% of TCE intake excreted in urinary GSH conjugates after 48 hr compared with about 18–27% excreted in urinary TCA + TCOH. Taking the quotient of estimated TCA + TCOH excreted to the upper bound of GSH conjugates excreted gives a lower bound on this excretion ratio of 360–540 to 1, which is indeed lower than and hence consistent with the estimated excretion ratio of 3,300–7,200 to 1 reported by Bernauer et al. (1996).

DCVC is thought to be a critical intermediate in the fate of GSH conjugates of TCE. Although one potential fate of DCVC is detoxification via NAT, bioactivation by renal enzymes to a toxic form is a potential parallel pathway. Thus, data on detoxification do not capture the total flux through the GSH pathway and are not informative regarding the amount bioactivated (Lash et al. 2000a). It has been hypothesized that bioactivation is through the renal β-lyase metabolism of DCVC, producing reactive metabolites that may contribute to renal toxicity (Anders et al. 1988). *In vitro* studies exist that measure human β-lyase activity in the kidney (Lash et al. 1990), but recent *in vitro* data (Krause et al. 2003; Lash et al. 2003) indicate that flavin-containing monooxygenases (FMOs) also may be toxicologically important for the bioactivation of DCVC, particularly in the human kidney.

Moreover, DCVC may become available to the kidney for bioactivation in multiple ways, and thus far no attempt has been made to model these complex interorgan processes. GSH conjugates produced in the liver may be exported directly to the blood into systemic circulation or to the bile, where they can be reabsorbed through the gut. Although the liver is the primary site of GSH conjugation, most tissues, including the kidney, contain GSTs (Lash et al. 2000a), so the contribution to the kidney of circulating DCVG produced in the liver relative to local production of DCVG is uncertain. *In vitro* studies (Cummings et al. 2000a, 2000b; Cummings and Lash 2000) have reported GSH conjugation of TCE in rat and human kidney cells, suggesting a role for local metabolism. This work has also identified several GST isoforms (mostly α class) in rat kidney cells and reported measurable activity toward TCE for those GSTs. Hissink et al. (2002) examined GSTs isolated from human liver and placenta and rat liver and kidney and found activity of μ-class GSTs but no detectable activity of α- or θ-class GSTs. Some *in vitro* data also exist on competition between TCE oxidation and conjugation (Lash et al. 1999a). GST activity was found not to diminish TCE oxidation, but CYP-mediated oxidation substantially diminished conjugation. The impact of variability in the GST pathway among humans was also evaluated in this *in vitro* study, but as mentioned above, extrapolating this variability to the *in vivo* scenario involves substantial uncertainty.

### Other extrahepatic metabolism

Although it is generally thought that the liver is the major site of TCE metabolism, CYPs, GSTs, and other metabolizing enzymes are distributed at varying levels of activity throughout other tissues (Lash et al. 2000a). Although extrahepatic metabolism may not contribute significantly to overall mass balance (Lash et al. 2000a), it may be important locally in terms of the toxicologic effects from *in situ* production of metabolites. In addition to the kidney, two potentially important sites are the lung and the male reproductive system.

As discussed by Green (2000), the oxidative pathway of TCE metabolism in mouse lung Clara cells is hypothesized to be responsible for the accumulation of CHL in mouse lungs, leading to cytotoxicity [see also Odum et al. (1992)]. Forkert and colleagues had previously reported cytotoxicity in mouse lung Clara cells from TCE exposure (Forkert and Birch 1989; Forkert and Forkert 1994; Forkert et al. 1985). Boers et al. (1999) reported the number of Clara cells in the human lung and indicated that Clara cells contribute substantially to cell renewal and are important in the development of lung adenocarcinoma in humans. Green (2000) suggested that although the activity of enzymes is lower in the lung as a whole than in the liver, the activity of CYP in the lung appears to be relatively higher than the activity of enzymes involved in clearing CHL and TCOH [believed to be alcohol dehydrogenase (ADH) and uridine diphosphate-glucuronosyltransferase (UGT)]. Hence, these two metabolites may accumulate in the mouse lung and lead to toxicity. Green (2000) suggests that such a mechanism in mice may not be relevant to humans because there is little CYP2E1 activity in the human lungs as a whole. However, metabolic activity from whole lungs may give misleading results because of the variety of cell types in which high activity in a few may be diluted by others with low activity, and the activities of the relevant enzymes for either CHL production or clearance in particular cell types have not been examined to date. In addition the relative contribution between local CHL production and circulating CHL (or CH), which has been measured in high-dose TCE exposures in mice (Abbas and Fisher 1997; Greenberg et al. 1999; Prout et al. 1985) and rats (Prout et al. 1985), has not been quantified.

Reports of TCE exposure affecting the male reproductive system, including the observation of Leydig cell tumors in rats exposed to TCE (Maltoni et al. 1986, 1988), have led to the investigation of metabolism and toxicity of TCE and its metabolites in the male reproductive system. Forkert et al. (2002, 2003) report several studies that indicate TCE oxidative metabolism occurs in the male reproductive tract. They detected CYP2E1 activity in the epididymal epithelium and testicular Leydig cells in mice, monkeys, and humans. Analysis of seminal fluid from eight human subjects diagnosed with clinical infertility and exposed to TCE occupationally was also performed and showed the presence of TCE, CHL, and TCOH in all eight subjects, DCA in two subjects, and TCA in one subject. TCA and/or TCOH were identified in urine samples from only two subjects. Although the lack of detailed exposure information limits the use of these data for development of a quantitative pharmacokinetic understanding, this evidence is qualitatively informative regarding the potential for local metabolism of TCE in the male reproductive tract.

## PBPK Modeling of TCE and Its Metabolites

TCE has an extensive number of both *in vivo* pharmacokinetic and PBPK modeling studies [summarized in Supplemental Material, Tables S-1 and S-2 (http://www.ehponline.org/members/2006/8691/suppl.pdf)]. Models designed for risk assessment applications have focused on descriptions of both TCE and major oxidative metabolites TCA, TCOH, and TCOG. Most of these models were extensions of the models developed by Fisher and co-workers (Allen and Fisher 1993; Fisher et al. 1991) in rats, mice, and humans. These models were based on a Ramsey and Andersen (1984) structure with perfusion-limited tissue compartments and equilibrium gas exchange, saturable Michaelis-Menten kinetics for metabolism, and lumped volumes for the major circulating oxidative metabolites TCA and TCOH. Fisher and co-workers updated their models with new *in vivo* and *in vitro* experiments performed in mice (Abbas and Fisher 1997; Greenberg et al. 1999) and human volunteers (Fisher et al. 1998) and summarized their findings in Fisher (2000). Clewell et al. (2000) did not include the updated Fisher data but did use a wider set of *in vivo* and *in vitro* mouse, rat, and human data than previous models. In addition Clewell et al. (2000) added EHR of TCOG and pathways for local oxidative metabolism in the lung and GST metabolism in the liver. Finally, Bois (2000a, 2000b) performed reestimations of PBPK model parameters for the Fisher and Clewell models using a Bayesian population approach (e.g., Gelman et al. 1996).

As discussed by Rhomberg (2000), using the models of Fisher (2000), Clewell et al. (2000), and Bois (2000a, 2000b) for cross-species extrapolation of rodent cancer bioassays led to sometimes substantially different quantitative results. One important difference is that model calibrations were based on different subsets of the database [summarized in Supplemental Material, Tables S-1 and S-2 (http://www.ehponline.org/members/2006/8691/suppl.pdf)]. The Clewell model was based primarily on a variety of data published before 1995, the Fisher models were based primarily on new studies conducted by Fisher and co-workers, as described above, and the Bois reestimations added to the Clewell data set but did not include some of the new Fisher data. In addition the Clewell model differed structurally in its use of single-compartment, volume-of-distribution models for metabolites compared with the Fisher models’ use of multiple physiologic compartments. Also, the Clewell model but not the Fisher models included EHR of TCOH/TCOG (although reabsorption was set to zero in some cases). Finally, the Bayesian statistical analysis used by Bois led to some differences in parameter estimates because all parameters were allowed to vary simultaneously compared with only a select few.

Given all these differences, it is not surprising that the different models led to different quantitative results. Even among the Fisher models themselves, Fisher (2000) noted inconsistencies, including differing estimates for metabolic parameters between mouse gavage and inhalation experiments. Possible explanations for these inconsistencies include the impact of corn oil vehicle use during gavage (Staats et al. 1991) and the impact of a decrease in ventilation rate in mice due to sensory irritation during the inhalation of solvents (e.g., Stadler and Kennedy 1996).

Throughout 2004 the U.S. EPA and the U.S. Air Force jointly sponsored an integration of the Fisher, Clewell, and Bois modeling efforts (Clewell et al. 2004). In brief, a single interim model structure combining features from both the Fisher and Clewell models was developed and used for all three species of interest (mice, rats, and humans). An effort was made to combine structures in a manner as simple as possible; the evaluation of most alternative structures was left for future work. However, species- and dose-dependent TCA plasma binding was implemented, although only the *in vitro* study of Lumpkin et al. (2003) was used as parameter inputs. A hierarchical Bayesian population analysis similar to the Bois (2000a, 2000b) analyses was performed on the revised model with a cross-section of the combined database of kinetic data to provide estimates of parameter uncertainty and variability (Hack et al. 2004, in press). Particular attention was given to using data from each of the different efforts, but because of time and resource constraints, a combined analysis of all data was not performed. The results from this effort suggested that a single model structure could provide reasonable fits to a variety of data evaluated for TCE and its major oxidative metabolites TCA, TCOH, and TCOG. However, in many cases, different parameter values—particularly for metabolism—were required for different studies, indicating significant interindividual or interexperimental variability. In addition it was concluded that dosimetry of DCA, conjugative metabolites, and metabolism in the lung remained highly uncertain (Clewell et al. 2004).

Although recent PBPK modeling studies have attempted to integrate different data sets, several research needs can be suggested for future work. Typically, PBPK models predict the concentration of chemicals at the target organ, making it possible to start linking dose metrics with pharmacodynamic effects. Caldwell and Keshava (2006) have reviewed additional factors that modulate the MOA of TCE that could be correlated with an appropriate dose metric predicted from a PBPK model. In addition a generalized PBPK model structure can be developed that integrates a larger fraction of the *in vivo* data sets in the published literature [summarized in Supplemental Material, Tables S-1 and S-2 (http://www.ehponline.org/members/2006/8691/suppl.pdf)]. Finally, with additional effort PBPK modeling can be applied to TCE in chemical mixtures, taking into account changes in metabolism induced by TCE itself, other solvents, disinfection by-products, and ethanol.

## Conclusions

Studies of the pharmacokinetics of TCE and its metabolites have been conducted for more than 30 years. Many early PBPK modeling efforts provided only a description of TCE itself and did not include any metabolites. Such models are still used for particular applications such as neurotoxicology (Boyes et al. 2005; Simmons et al. 2002), understanding the tissue distribution of TCE (Albanese et al. 2002; Keys et al. 2003), or assessing pharmacokinetic interactions of mixtures (Dobrev et al. 2001). Models that include metabolite descriptions have focused primarily on TCE and its major circulating oxidative metabolites TCA and TCOH, and its glucuronide, with some attempt at quantifying other metabolic pathways with potential toxicological importance (Clewell et al. 2000; Fisher 2000). Finally, a number of recent modeling efforts have sought to integrate the body of existing pharmacokinetic information on TCE and its metabolites (Bois 2000a, 2000b; Clewell et al. 2004; Hack et al. 2004, in press), highlighting both apparent variability and some inconsistencies among studies across the database.

For risk assessment it is particularly important to characterize the impact of both variability (i.e., irreducible heterogeneity) and uncertainty (i.e., lack of knowledge) on toxicologically relevant dosimetry. Although PBPK modeling in risk assessment is intended to provide more accurate estimates of dose relative to default procedures, it should be recognized that in some cases, rigorous analysis of PBPK models may reveal uncertainties not previously considered or of greater magnitude than that which is assumed under default procedures. Even for TCE, TCA, and TCOH, a number of structural hypotheses remain to be tested, and some inconsistencies can be better understood. However, a large database of information exists on the pharmacokinetics of these three chemicals in mice, rats, and humans, and statistical methods such as hierarchical Bayesian population modeling and computation tools such as Markov chain Monte Carlo analyses are now available to conduct complex analyses of parameter and model sensitivity and uncertainty [e.g., see discussion in Bernillon and Bois (2000)]. Therefore, even if these remaining uncertainties cannot yet be resolved, it appears feasible, at least in principle, to characterize their effect on risk assessment quantitatively while at the same time providing insight into potential experimental studies that may help reduce these uncertainties.

Furthermore, given the number of studies conducted in different individuals, characterization of interindividual variability for TCE, TCA, and TCOH pharmacokinetics also appears feasible. Although extrapolation from human volunteers to the broader human population presents some additional uncertainties, in some cases *in vitro* data may be useful to inform this inference. Thus, the challenge for TCE, TCA, and TCOH is primarily in implementation—that is, the practical difficulties of analyzing a large database of information against a number of different hypotheses. Unfortunately, the pharmacokinetics database is much more sparse, particularly in terms of reliable *in vivo* data, for several metabolites of toxicological interest, including DCA, local production and clearance of CHL in the lung, and conjugative metabolites. Thus, the challenge in these cases is whether, given the current lack of ability to verify pharmacokinetics *in vivo*, either *in vitro* data or better calibrated (but potentially less toxicologically relevant) dose surrogates exist that could provide sufficiently reliable information for application to risk assessment.
