# Supplementary material for: Issues in the Pharmacokinetics of Trichloroethylene and Its Metabolites
Source: Environ Health Perspect. 2006 May 9;114(9):1450–6. doi: 10.1289/ehp.8691 (PMC1570093; doi:10.1289/ehp.8691)
Supplement: Supplemental Figures and Tables [file ehp0114-001450s1.pdf]

Supplemental Material

for

Issues in the Pharmacokinetics of Trichloroethylene and Its Metabolites

Weihsueh A. Chiu, Miles S. Okino, John C. Lipscomb, and Marina V. Evans

Supplemental Material

This manuscript has focused on research efforts published since the 2000 state-of-the science monograph, including past research relevant to those efforts. These supplemental materials provide a comprehensive description of pharmacokinetic studies on TCE and its metabolites and PBPK models for TCE.

Supplemental Table S-1 "Summary of *in vivo* studies of trichloroethylene (TCE) pharmacokinetics" includes descriptions of the pharmacokinetic studies on TCE and its metabolites in mice, rats, and humans. The dosing scenarios are listed along with the measurements taken in each study. The table also indicates the PBPK models that used multiple data sets for calibration and evaluation. The PBPK models that used only a single data set collected by the authors are not included.

Supplemental Table S-2 "Summary of PBPK models of trichloroethylene (TCE) and metabolites" provides summaries of published PBPK models. The species, exposure routes, compartments, and metabolites simulated in the models are listed for comparison. Many of the PBPK models have been used for subsequent analyses, although those efforts are not detailed in this table.

Table S-1. Summary of *in vivo* studies of trichloroethylene (TCE) pharmacokinetics

			Exposures		Reported Measurements				Physiologically based pharmacokinetic models analyzing multiple datasets											
Reference	Species (Strain, if appl.)	Sex (M, F, M+F)	TCE	Other (if appl.)	TCE	TCA	TCOH/TCOG[a]	Other	Allen and Fisher 1993	Bois 2000a	Bois 2000b	Clewell et al. 2000	Clewell et al. 2004	Fisher 2000	Green-berg et al. 1999	Hack et al. 2004	Keys et al. 2003	Lapare et al. 1995	Stenner et al. 1998	Thrall and Poet 2000
Human studies																				
Bartonicek 1962	Human	M+F	Inhalation		exhaled air	plasma, red cells, urine, sweat, saliva	urine, sweat, saliva	TCOH+TCA in feces												
Bernauer et al.1996	Human	M	Inhalation		--	--	--	TCA+TCOH in urine, NADCVC in urine				X	X			X				
Bloemen et al.2001	Human	M	Inhalation		uptake	urine	urine	NADCVC in urine												
Ertle et al.1972	Human	M	Inhalation	CH oral	--	urine	blood, urine													
Fernandez et al.1977	Human	M	Inhalation		alveolar air	urine	urine	--												
Fisher et al.1998	Human	M+F	Inhalation		exhaled air, blood	blood, urine	blood (H), urine (G)	CH in blood, DCA plasma		X			X	X		X[b]				
Kimmerle and Eben 1973b	Human	M+F	Inhalation		exhaled air, blood	urine	blood, urine	CH in blood												
Lapare et al.1995	Human	M+F	Inhalation		alveolar air, blood	urine	urine	--										X		
Lash et al.1999	Human	M+F	Inhalation		--	--	--	DCVG in blood												
Monster et al.1976	Human	M	Inhalation		exhaled air, blood	blood, urine	blood, urine	--	X	X[c]	X			X				X		
Monster et al.1979	Human	M	Inhalation		exhaled air, blood	blood, urine	blood, urine	--	X	X[c]	X	X	X	X						
Muller et al.1972	Human	ns	Inhalation		--	plasma, urine	blood, urine	--												
Muller et al.1974	Human	M	Inhalation	CH, TCA, TCOH oral	alveolar air, blood	plasma, urine	blood, urine	--	X	X[c]	X	X	X	X						
Muller et al.1975	Human	M	Inhalation	Ethanol oral	alveolar air, blood	plasma, urine	blood, urine	ethanol, acetaldehyde in blood	X	X[c]	X	X	X	X						
Paykoc et al.1945	Human	ns	--	TCA iv	--	blood, plasma, urine	--	--												
Poet et al.2000	Human	M+F	Dermal		exhaled air	--	--	--												
Sato et al.1977	Human	M	Inhalation		alveolar air, blood	urine	urine	--												
Sato and Nakajima 1978	Human	M	Dermal		alveolar air, blood	urine	urine	--												
Soucek and Vlachova 1960	Human	M+F	Inhalation	glucose and insulin iv (1 subject)	retention	urine	urine	MCA in urine												
Stewart et al.1970	Human	ns	Inhalation		alveolar air	urine	urine	--	X	X[c]	X	X	X	X		X				
Vesterberg and Astrand 1976[d]	Human	M	Inhalation		alveolar air, arterial and venous blood	blood, urine	blood, urine	--												
Mouse studies																				
Abbas et al.1996	Mouse (B6C3F1)	M	--	CH iv		blood	blood (H,G)	CH in blood, AUC CH in liver, DCA in blood		X										
Abbas and Fisher 1997	Mouse (B6C3F1)	M	Oral (corn oil)		blood, liver, fat, kidney	blood, liver, kidney, urine	blood (H,G), liver (H,G), lung (H), kidney (H,G), urine (G)	CH in blood, liver, lung, kidney; DCA in blood, liver, kidney		X			X	X			X			
Barton et al.1999	Mouse (B6C3F1)	M	--	DCA iv and oral (aqueous)	--	--	--	DCA in blood												
Birner et al.1993	Mouse (NMRI)	M+F	Gavage (ns)		--	urine	--	NADCVC in urine												
Fisher and Allen 1993	Mouse (B6C3F1)	M+F	Gavage (corn oil)		blood	plasma	--	--		X[c]	X			X						
Fisher et al.1991	Mouse (B6C3F1)	M+F	Inhalation		blood, closed chamber	plasma	--	--		X[c]	X	X	X	X	X	X				
Green and Prout 1985	Mouse (B6C3F1)	M	Gavage (corn oil)		exhaled air	urine	urine	%dose as urine, feces, CO2												
Greenberg et al.1999	Mouse (B6C3F1)	M	Inhalation		exhaled air, blood, liver, kidney, lung, fat	blood, liver, kidney, lung	blood (H,G), liver (H,G), lung (H), kidney (H,G)	CH in blood, liver, lung, kidney; DCA in blood, liver, kidney					X	X	X	X				
Larson and Bull 1992a	Mouse (B6C3F1)	M	--	DCA, TCA oral (aqueous)	--	plasma, urine	plasma, urine	%dose as urine, feces, CO2; DCA in plasma, urine; TDAA, MCA in urine		X[c]	X									
Larson and Bull 1992b	Mouse (B6C3F1)	M	Oral (aqueous)		blood	blood	blood (H)	DCA in blood		X[c]	X									
Merdink et al.1998	Mouse (B6C3F1)	M	iv	CH iv	--	blood	blood (H)	CH in blood												
Prout et al.1985	Mouse (B6C3F1, Swiss)	M	Gavage (corn oil)		exhaled air, blood	blood	blood (--)	CHL in blood, %dose as urine, feces, CO2, carcass		X[c]	X	X	X			X				
Templin et al.1993	Mouse (B6C3F1)	M	Oral (aqueous)	TCA oral	blood	blood	blood (H, G)	DCA in blood		X	X	X	X			X				

Table S-1. Summary of *in vivo* studies of trichloroethylene (TCE) pharmacokinetics (cont.)

Reference	Species (Strain, if appl.)	Sex (M, F, M+F)	Exposures: TCE	Exposures: Other (if appl.)	Reported Measurements: TCE	Reported Measurements: TCA	Reported Measurements: TCOH/TCOG[a]	Reported Measurements: Other	PBPK models analyzing multiple datasets: Allen and Fisher 1993	Bois 2000a	Bois 2000b	Clewell et al. 2000	Clewell et al. 2004	Fisher 2000	Green-berg et al. 1999	Hack et al. 2004	Keys et al. 2003	Lapare et al. 1995	Stenner et al. 1998	Thrall and Poet 2000
Rat studies																				
Andersen et al.1987	Rat (Fisher)	M	Inhalation		closed chamber	--	--	--					X			X				
Barton et al.1995	Rat (SD)	M	Inhalation		closed chamber	--	--	--												
Bernauer et al.1996	Rat (Wistar)	M	Inhalation		--	--	--	TCA+TCOH in urine, NADCVC in urine				X	X			X				
Birner et al.1993	Rat (Wistar, Fisher 344)	M+F	Gavage (ns)		--	urine	--	NADCVC in urine												
Dallas et al.1991	Rat (SD)	M	Inhalation		exhaled air, blood	--	--	--												
D'Souza et al.1985	Rat (SD)	M	iv, oral (aqueous)		blood	--	--	--												
Fisher et al.1991	Rat (Fisher 344)	M+F	Inhalation		blood	plasma	--	--			X	X	X			X				X
Green and Prout 1985	Rat (Osborne-Mendel)	M	Gavage (corn oil)	TCA gavage (aqueous)	exhaled air	urine	urine	%dose as urine, feces, CO2												
Hissink et al.2002	Rat (Wistar)	M	Gavage (corn oil), iv		blood	--	--	%dose as urine, feces, expired air; radioactivity in organs												
Jakobson et al.1986	Rat (SD)	F	Inhalation	Pretreatment (oral) with ethanol, other chemicals	blood	--	--	--												
Kaneko et al.1994	Rat (Wistar)	M	Inhalation	Pretreatment (oral) with ethanol	blood	urine	urine	--												
Keys et al.2003	Rat (SD)	M	Inhalation, oral (aqueous), ia		blood, liver, fat, kidney, lung, muscle, GI, brain, heart,	--	--	--									X			
Kimmerle and Eben 1973a	Rat (Wistar)	M	Inhalation		exhaled air, blood, fat, brain, liver, kidney, spleen, heart	urine	blood, urine	CH in blood												
Larson and Bull 1992a	Rat (Fisher 344)	M	--	DCA, TCA oral (aqueous)	--	plasma, urine	plasma, urine	%dose as urine, feces, CO2; DCA in plasma, urine; TDAA, MCA in urine			X	X	X			X				
Larson and Bull 1992b	Rat (SD)	M	Oral (aqueous)		blood	blood	blood (H)	DCA in blood			X								X	
Lee et al.1996	Rat (SD)	M	Arterial, venous, portal, stomach injections		blood	--	--	--												
Lee et al.2000	Rat (SD)	M	Stomach injection	pretreatment (ia) with p-nitrophenol	blood	--	--	--												
Merdink et al.1999	Rat (Fisher 344)	M	--	CH, TCOH iv	--	blood, bile	blood (H, G), bile (G)	CH in blood, bile											X	
Poet et al.2000	Rat (Fisher 344)	M	Dermal		chamber	--	--	--												
Prout et al.1985	Rat (Osborne-Mendel, Wistar)	M	Gavage (corn oil)		exhaled air, blood	blood	blood (--)	CHL in blood, %dose as urine, feces, CO2, carcass			X	X	X			X				
Saghir et al.2002	Rat (Fisher 344)	M	--	DCA iv, oral (aqueous)	--	--	--	DCA in plasma												
Simmons et al.2002	Rat (Long-Evans)	M	Inhalation		closed chamber, blood, liver, brain, fat	--	--	--												
Stenner et al.1997	Rat (Fisher 344)	M	intraduodenal	TCOH, TCA iv	--	blood, bile	blood, bile	--											X	
Templin et al.1995	Rat (Fisher 344)	M	Oral (aqueous)		blood	blood	blood, bile (H,G)	--			X	X							X	
Thrall et al.2000	Rat (Fisher 344)	M	iv	with tolune	exhaled breath, closed chamber	--	--	--												X

Notes:
[a] Combined free+bound unless otherwise specified [(H) = free only, (G) = bound only, (H,G) = both, separately]
[b] Subset of individuals used
[c] Implicit through use of Bois-Clewell posteriors as priors
[d] Summary of data reported in Astrand and Ovrum (1976) and Vesterberg et al. (1976)
ns= not specified

Abbreviations: AUC = area under the concentration curve; CH = chloral hydrate; CHL = chloral; CO2 = carbon dioxide; DCA = dichloroacetic acide; DCVC = *S*-dichlorovinyl-L-cysteine; DCVG = *S*-dichlorovinyl glutathione; iv = intravenous; MCA = monochloroacetic acid; NADCVC = *N*-acetyl DCVC; SD = Sprague-Dawley; TCA = trichloroacetic acid; TCE = trichloroethylene; TCOH = trichloroethanol; TCOG = TCOH glucuronide; TDAA = thiodiacetic acid

Table S-2: Summary of physiologically based pharmacokinetic models of trichloroethylene (TCE) and metabolites

			Exposure scenarios		TCE compartments[a]								TCA compartments[b]						Other metabolites/compartments				
Reference	Species (Strain, if appl.)	Sex	TCE	Other	fat	GI	kidney	liver	lung	rapidly perfused	slowly perfused	other	body	kidney	liver	lung	Vdist	other	TCOH	TCOG	DCA	CHL/ CH	DCVC
Abbas and Fisher 1997	Mice B6C3F1	Male	oral		x	2	x	x	x	x	x		x	x	x	x			same as TCA	same as TCA	same as TCA	same as TCA	
Allen and Fisher 1993	Human		inhalation		x			x	x	x	x						x						
Andersen et al. 1987	Rats F344	Male	inhalation	inhaled TCE with DCE	x			x	x	x	x												
Barton et al. 1999	Mice B6C3F1	Male		chronic oral DCA, acute iv DCA																	body, GI, liver		
Clewell et al. 2000	Mice, Rat, Human		inhalation and oral		x	2		x	x	x	x	tracheo-bronchial					x		Vdist	Vdist and bile	Vdist	lung	Vdist
Clewell et al. 2004 [c]	Mice, Rat, Human		inhalation, iv, oral		x	2		x	x	x	x	tracheo-bronchial	x		x				Vdist	Vdist and bile	Vdist	lung	Vdist
Dallas et al. 1991	Rat SD	Male	inhalation		x			x	x	x	x												
Fernandez et al. 1977	Human	Male	inhalation		x			x	x	x	x						x		Vdist			Vdist	
Fisher et al. 1989	Rat F344	Female, pregnant	inhalation and oral	iv TCA	x	x		x	x	x	x	fetus, mammary tissue, placenta	x					placenta, fetus					
Fisher et al. 1990	Rat F344; adult and	Female, lactating	inhalation and oral		x	x		x	x	x	x	milk	x					mammary tissue, milk					
Fisher et al. 1991	Mice B6C3F1; Rats F344	Female and Male	inhalation		x			x	x	x	x						x						
Fisher et al. 1998	Human	Female and Male	inhalation		x		x	x	x	x	x		x	x	x	x			same as TCA	Vdist			
Greenberg et al. 1999	Mice B6C3F1	Male	inhalation		x		x	x	x	x	x		x	x	x	x			same as TCA	same as TCA	same as TCA	same as TCA	
Keys et al. 2003	Mice B6C3F1; Rats SD	Male	inhalation, ia injection, oral		2	x	x	2	x	x	x	brain, heart, spleen											
Keys et al. 2004	Mice B6C3F1; Rats F344	Male		chronic oral DCA, acute iv DCA																	kidney, liver, rapidly perf,		
Koizumi 1989	Rats OM; Human	Male	inhalation and oral		x			x	x	x	x												
Lapare et al. 1995	Humans		inhalation		x	x		x	x	x	x						x		Vdist				
Poet et al. 2000	Rat F344, Humans	Male rats; Female	dermal		x			x	x	x	x	skin											
Sato et al. 1991	Human	Female and Male	inhalation		x	x		x	x	x	x	muscle					x		Vdist				
Simmons et al. 2002	Rats long-evans	Male	inhalation		x			x	x	x	x	brain											
Stenner et al. 1998	Rats F344	Male	oral	iv CH, TCA, TCOH	x	x	x	x	x	x	x				x			fat, GI, rapid perf, slow perf, bile	fat, GI, rapid perf, slow perf	GI, rapid perf, slow perf, bile			
Thrall and Poet 2000	Rats F344	Male	iv, ip injection	iv TCE with toluene	x			x	x	x	x												

Notes:
[a] "2" indicates a 2-compartment organ
[b] Vdist=volume of distribution
[c] Development of a physiologically-based pharmacokinetic model of trichloroethylene and its metabolites for use in risk assessment. Report prepared for U.S. Air Force. Available from: http://www.tera.org/vera/TCE/TCE%20PBPK%20harmonization%20document.

Abbreviations: CH = chloral hydrate; CHL = chloral; DCA = dichloroacetic acide; DCVC = *S*-dichlorovinyl-L-cysteine; ia = intraarterial; ip = intraperitoneal; iv = intravenous; GI = gastro-intestinal; OM = Osborne-Mendel; SD = Sprague-Dawley; TCA = trichloroacetic acid; TCE = trichloroethylene; TCOH = trichloroethanol; TCOG = TCOH glucuronide

References for Supplemental Materials

Abbas R, Fisher JW. 1997. A physiologically based pharmacokinetic model for trichloroethylene and its metabolites, chloral hydrate, trichloroacetate, dichloroacetate, trichloroethanol, and trichloroethanol glucuronide in B6C3F1 mice. Toxicol Appl Pharmacol 147:15-30.

Abbas R, Seckel CS, Kidney JK, Fisher JW. 1996. Pharmacokinetic analysis of chloral hydrate and its metabolism in B6C3F1 mice. Drug Metab Dispos 24(12):1340-1346.

Allen BC, Fisher JW. 1993. Pharmacokinetic modeling of trichloroethylene and trichloroacetic acid in humans. Risk Anal 13:71-86.

Anders MW, Lash L, Dekant W, Elfarra AA, Dohn DR. 1988. Biosynthesis and biotransformation of glutathione S-conjugates to toxic metabolites. Crit Rev Toxicol 18:311-341.

Andersen ME, Gargas ML, Clewell HJ, Severyn KM. 1987. Quantitative evaluation of the metabolic interactions between trichloroethylene and 1,1-dichloroethylene in vivo using gas uptake methods. Toxicol Appl Pharmacol 89:149-157.

Astrand I, Ovrum P. 1976. Exposure to trichloroethylene I. Uptake and distribution in man. Scand J Work Environ Health 2(4):199-211.

Barton HA, Creech JR, Godin CS, Randall GM, Seckel CS. 1995. Chloroethylene mixtures: Pharmacokinetic modeling and in vitro metabolism of vinyl chloride, trichloroethylene, and trans-1,2-dichloroehtylene in the rat. Toxicol Appl Pharmacol 130:237-247.

Barton HA, Bull R, Schultz I, Andersen ME. 1999. Dichloroacetate (DCA) dosimetry: interpreting DCA-induced liver cancer dose response and the potential for DCA to contribute to trichloroethylene-induced liver cancer. Toxicol Lett 106(1):9-21.

Bartonicek V. 1962. Metabolism and excretion of trichloroethylene after inhalation by human subjects. Br J Ind Med 19:134-142.

Bernauer U, Birner G, Dekant W, Henschler D. 1996. Biotransformation of trichloroethylene: dose-dependent excretion of 2,2,2-trichlor-metabolites and mercapturic acids in rats and humans after inhalation. Arch Toxicol 70(6):338-346.

Birner G, Vamvakas S, Dekant W, Henschler D. 1993. Nephrotoxic and genotoxic N-acetyl-S-dichlorovinyl-L-cysteine is a urinary metabolite after occupational 1,1,2-trichloroethylene exposure in humans: implications for the risk of trichloroethylene exposure. Environ Health Perspect 99:281-284.

Bloemen LJ, Monster AC, Kezic S, Commandeur JN, Veulemans H, Vermeulen NP, et al. 2001. Study on the cytochrome P-450- and glutathione-dependent biotransformation of trichloroethylene in humans. Int Arch Occup Environ Health 74(2):102-108.

Clewell HJ, Covington TR, Fisher JW, Chiu WA, Lipscomb JC. 2004. Development of a physiologically-based pharmacokinetic model of trichloroethylene and its metabolites for use in risk assessment. Report prepared for the U.S. Air Force. Available: http://www.tera.org/vera/TCE/TCE%20PBPK%20harmonization%20document.pdf [accessed September 20, 2005].

Clewell HJ, Gentry PR, Covington TR, Gearhart JM. 2000. Development of a physiologically based pharmacokinetic model of trichloroethylene and its metabolites for use in risk assessment. Environ Health Perspect 108(suppl 2):283-305.

Dallas CE, Gallo JM, Ramanathan R, Muralidhara S, Bruckner JV. 1991. Physiologic pharmacokinetic modeling of inhaled trichloroethylene in rats. Toxicol Appl Pharmacol 110:303-314.

D'Souza RW, Bruckner JV, Feldman S. 1985. Oral and intravenous trichloroethylene pharmacokinetics in the rat. J Toxicol Environ Health 15:587-601.

Ertle T, Henschler D, Muller G, Spassowski M. 1972. Metabolism of trichloroethylene in man. I. The significance of trichloroethanol in long-term exposure conditions. Arch Toxicol 29:171-188.

Fernandez JG, Droz PO, Humbert BE, Caperos JR. 1977. Trichloroethylene exposure: simulation of uptake, excretion, and metabolism using a mathematical model. Br J Ind Med 34(1):43-55.

Fisher JW, Allen BC. 1993. Evaluating the risk of liver cancer in humans exposed to trichloroethylene using physiological models. Risk Anal 13(1):87-95.

Fisher JW, Gargas ML, Allen BC, Andersen ME. 1991. Physiologically based pharmacokinetic modeling with trichloroethylene and its metabolite, trichloroacetic acid, in the rat and mouse. Toxicol Appl Pharmacol 109:183-195.

Fisher JW, Mahle DA, Abbas R. 1998. A human physiologically based pharmacokinetic model for trichloroethylene and its metabolites, trichloroacetic acid and free trichloroethanol. Toxicol Appl Pharmacol 152:339-359.

Fisher JW, Whittaker TA, Taylor DH, Clewell HJ, Andersen ME. 1989. Physiologically based pharmacokinetic modeling of the pregnant rat: a multiroute exposure model for trichloroethylene and its metabolite, trichloroacetic acid. Toxicol Appl Pharmacol 99:395-114.

Fisher JW, Whittaker TA, Taylor DH, Clewell HJ, Andersen ME. 1990. Physiologically based pharmacokinetic modeling of the lactating rat and nursing pup: a multiroute exposure model for trichloroethylene and its metabolite, trichloroacetic acid. Toxicol Appl Pharmacol 102:497-513.

Green T, Prout MS. 1985. Species differences in response to trichloroethylene. II. Biotransformation in rats and mice. Toxicol Appl Pharmacol 79(3):401-411.

Greenberg MS, Burton GA, Fisher JW. 1999. Physiologically based pharmacokinetic modeling of inhaled trichloroethylene and its oxidative metabolites in B6C3F1 mice. Toxicol Appl Pharmacol 154:264-278.

Hissink EM, Bogaards JJP, Freidig AP, Commandeur JNM, Vermeulen NPE, van Bladeren PJ. 2002. The use of in vitro metabolic parameters and physiologically based pharmacokinetic (PBPK) modeling to explore the risk assessment of trichloroethylene. Environ Toxicol Pharm 11:259-271.

Jakobson I, Holmberg B, Ekner A. 1986. Venous blood levels of inhaled trichloroethylene in female rats and changes induced by interacting agents. Acta Pharmacol Toxicol (Copenh) 59(2):135-143.

Kaneko T, Wang PY, Sato A. 1994. Enzymes induced by ethanol differently affect the pharmacokinetics of trichloroethylene and 1,1,1-trichloroethane. Occup Environ Med 51:113-119.

Keys DA, Bruckner JV, Muralidhara S, Fisher JW. 2003. Tissue dosimetry expansion and cross-validation of rat and mouse physiologically based pharmacokinetic models for trichloroethylene. Toxicol Sci 76(1):35-50.

Keys DA, Schultz IR, Mahle DA, Fisher JW. 2004. A quantitative description of suicide inhibition of dichloroacetic acid in rats and mice. Toxicol Sci 82(2):381-393.

Kimmerle G, Eben A. 1973a. Metabolism, excretion and toxicology of trichloroethylene after inhalation. 1. Experimental exposure on rats. Arch Toxikol 30(2):115-126.

Kimmerle G, Eben A. 1973b. Metabolism, excretion and toxicology of trichloroethylene after inhalation. 2. Experimental human exposure. Arch Toxikol 30(2):127-138.

Koizumi A. 1989. Potential of physiologically based pharmacokinetics to amalgamate kinetic data of trichloroethylene and tetrachloroethylene obtained in rats and man. Br J Ind Med 46:239-249.

Lapare S, Tardif R, Brodeur J. 1995. Effect of various exposure scenarios on the biological monitoring of organic solvents in alveolar air. II. 1,1,1-trichloroethane and trichloroethylene. Int Arch Occup Environ Health 67(6):375-394.

Larson JL, Bull RJ. 1992a. Metabolism and lipoperoxidative activity of trichloroacetate and dichloroacetate in rats and mice. Toxicol Appl Pharmacol 115(2):268-277.

Larson JL, Bull RJ. 1992b. Species differences in the metabolism of trichloroethylene to the carcinogenic metabolites trichloroacetate and dichloroacetate. Toxicol Appl Pharmacol 115(2):278-285.

Lash LH, Putt DA, Brashear WT, Abbas R, Parker JC, Fisher JW. 1999. Identification of S-(1,2-dichlorovinyl)glutathione in the blood of human volunteers exposed to trichloroethylene. J Toxicol Environ Health 56(1):1-21.

Lee KM, Bruckner JV, Muralidhara S, Gallo JM. 1996. Characterization of presystemic elimination of trichloroethylene and its nonlinear kinetics in rats. Toxicol Appl Pharmacol 139(2):262-271.

Lee KM, Muralidhara S, White CA, Bruckner JV. 2000. Mechanisms of the dose-dependent kinetics of trichloroethylene: oral bolus dosing of rats. Toxicol Appl Pharmacol 164(1):55-64.

Merdink JL, Gonzalez-Leon A, Bull RJ, Schultz IR. 1998. The extent of dichloroacetate formation from trichloroethylene, chloral hydrate, trichloroacetate, and trichloroethanol in B6C3F1 mice. Toxicol Sci 45:33-41.

Merdink JL, Stenner RD, Stevens DK, Parker JC, Bull RJ. 1999. Effect of enterohepatic circulation on the pharmacokinetics of chloral hydrate and its metabolites in F344 rats. J Toxicol Environ Health A. 57(5):357-368.

Monster AC, Boersma G, Duba WC. 1976. Pharmacokinetics of trichloroethylene in volunteers, influence of workload and exposure concentration. Int Arch Occup Environ Health 38(2):87-102.

Monster AC, Boersma G, Duba WC. 1979. Kinetics of trichloroethylene in repeated exposure of volunteers. Int Arch Occup Environ Health 42(3-4):283-292.

Müller G, Spassovski M, Henschler D. 1972. Trichloroethylene exposure and trichloroethylene metabolites in urine and blood. Arch Toxicol 29:335-340.

Müller G, Spassovski M, Henschler D. 1974. Metabolism of trichloroethylene in man. II. Pharmacokinetics of metabolites. Arch Toxicol 32(4):283-295.

Müller G, Spassovski M, Henschler D. 1975. Metabolism of trichloroethylene in man. III. Interaction of trichloroethylene and ethanol. Arch Toxicol 33(3):173-189.

Paykoc ZV, Powell JF. 1945. The excretion of sodium trichloroacetate. J Pharmacol Exp Ther 85:289-293.

Poet TS, Corley RA, Thrall KD, Edwards JA, Tanoko H, Weitz KK, et al. 2000. Assessment of the percutaneous absorption of trichloroethylene in rats and humans using MS/MS real-time breath analysis and physiologically based pharmacokinetic modeling. Toxicol Sci 56(1):61-72.

Prout MS, Provan WM, Green T. 1985. Species differences in response to trichloroethylene. Toxicol Appl Pharmacol 79:389-400.

Saghir SA, Schultz IR. 2002. Low-dose pharmacokinetics and oral bioavailability of dichloroacetate in naïve and GST-zeta-depleted rats. Environ Health Perspect 110:757-763.

Sato A, Nakajima T. 1978. Differences following skin or inhalation exposure in the absorption and excretion kinetics of trichloroethylene and toluene. Br J Ind Med 35(1):43-49.

Sato A, Nakajima T, Fujiwara Y, Murayama N. 1977. A pharmacokinetic model to study the excretion of trichloroethylene and its metabolites after an inhalation exposure. Br J Ind Med 34(1):56-63.

Sato A, Endoh K, Kaneko T, Johanson G. 1991. A simulation study of physiological factors affecting pharmacokinetic behaviour of organic solvent vapors. Br J Ind Med 48:342-347.

Simmons JE, Boyes WK, Bushnell PJ, Raymer JH, Limsakun T, McDonald A, et al. 2002. A physiologically based pharmacokinetic model for trichloroethylene in the male Long-Evans rat. Toxicol Sci 69(1):3-15.

Soucek B, Vlachova D. 1960. Excretion of trichloroethylene metabolites in human urine. Br J Ind Med 17:60-64.

Stenner RD, Merdink JL, Fisher JW, Bull RJ. 1998. Physiologically-based pharmacokinetic model for trichloroethylene considering enterohepatic recirculation of major metabolites. Risk Anal 18(3):261-269.

Stenner RD, Merdink JL, Stevens DK, Springer DL, Bull RJ. 1997. Enterohepatic recirculation of trichloroethanol glucuronide as a significant source of trichloroacetic acid in the metabolism of trichloroethylene. Drug Metab Disp 25(5):529-535.

Stewart RD, Dodd HC, Gay HH, Erley DS. 1970. Experimental human exposure to trichloroethylene. Arch Environ Health 20(1):64-71.

Templin MV, Parker JC, Bull RJ. 1993. Relative formation of dichloroacetate and trichloroacetate from trichloroethylene in male B6C3F1 mice. Toxicol Appl Pharmacol 23(1):1-8.

Templin MV, Stevens DK, Stenner RD, Bonate PL, Tuman D, Bull RJ. 1995. Factors affecting species differences in the kinetics of metabolites of trichloroethylene. J Toxicol Environ Health 44:435-447.

Thrall KD, Poet TS. 2000. Determination of biokinetic interactions in chemical mixtures using real-time breath analysis and physiologically based pharmacokinetic modeling. J Toxicol Environ Health A. 59(8):653-670.

Vesterberg O, Astrand I. 1976. Exposure to trichloroethylene monitored by analysis of metabolites in blood and urine. J Occup Med 18(4):224-226.

Vesterberg O, Gorczak J, Krasts M. 1976. Exposure to trichloroethylene II. Metabolites in blood and urine. Scand J Work Environ Health 2(4):212-219.
